# Canadian Consensus Recommendations on the Management of *MET*-Altered NSCLC

**DOI:** 10.3390/curroncol28060386

**Published:** 2021-11-09

**Authors:** Parneet K. Cheema, Shantanu O. Banerji, Normand Blais, Quincy S.-C. Chu, Patrice Desmeules, Rosalyn A. Juergens, Natasha B. Leighl, Brandon S. Sheffield, Paul F. Wheatley-Price, Barbara L. Melosky

**Affiliations:** 1 Medical Oncology/Hematology, William Osler Health System, Brampton, ON L6R 3J7, Canada; 2 Faculty of Medicine, University of Toronto, Toronto, ON M5S 1A8, Canada; 3 CancerCare Manitoba Research Institute, Department of Medical Oncology, CancerCare Manitoba, University of Manitoba, Winnipeg, MB R3E 0V9, Canada; sbanerji@cancercare.mb.ca; 4 Department of Medicine, Centre Hospitalier de l’Université de Montréal, University of Montreal, Montreal, QC H2X 3E4, Canada; normand.blais.med@ssss.gouv.qc.ca; 5 Cross Cancer Institute, Alberta Health Services, Edmonton, AB T6G 1Z2, Canada; quincy.chu@albertahealthservices.ca; 6 Service d’Anatomopathologie et de Cytologie, Institut Universitaire de Cardiologie et de Pneumologie de Québec, Université Laval, Quebec City, QC G1V 0A6, Canada; patrice.desmeules@criucpq.ulaval.ca; 7 Department of Medical Oncology, Juravinski Cancer Centre, McMaster University, Hamilton, ON L8V 5C2, Canada; juergensr@hhsc.ca; 8 Princess Margaret Cancer Centre, University Health Network, Department of Medicine, University of Toronto, Toronto, ON M5S 1A8, Canada; natasha.leighl@uhn.ca; 9 Department of Laboratory Medicine, William Osler Health System, Brampton, ON L6R 3J7, Canada; brandon.s.sheffield@gmail.com; 10 Department of Medicine, The Ottawa Hospital Research Institute, The Ottawa Hospital, University of Ottawa, Ottawa, ON K1H 8L6, Canada; pwheatleyprice@toh.ca; 11 Department of Medical Oncology, BC Cancer-Vancouver Centre, Vancouver, BC V5Z 4E6, Canada; BMelosky@bccancer.bc.ca

**Keywords:** non-small cell lung cancer, *MET* exon 14 skipping mutations, *MET* amplification, *MET* inhibitors, EGFR resistance

## Abstract

In Canada, the therapeutic management of patients with advanced non-small cell lung cancer (NSCLC) with rare actionable mutations differs between provinces, territories, and individual centres based on access to molecular testing and funded treatments. These variations, together with the emergence of several novel mesenchymal-epithelial transition (*MET*) factor-targeted therapies for the treatment of NSCLC, warrant the development of evidence-based consensus recommendations for the use of these agents. A Canadian expert panel was convened to define key clinical questions, review evidence, discuss practice recommendations and reach consensus on the treatment of advanced *MET*-altered NSCLC. Questions addressed by the panel include: 1. How should the patients most likely to benefit from *MET-*targeted therapies be identified? 2. What are the preferred first-line and subsequent therapies for patients with *MET* exon 14 skipping mutations? 3. What are the preferred first-line and subsequent therapies for advanced NSCLC patients with de novo *MET* amplification? 4. What is the preferred therapy for patients with advanced epidermal growth factor receptor (*EGFR*)-mutated NSCLC with acquired *MET* amplification progressing on EGFR inhibitors? 5. What are the potential strategies for overcoming resistance to *MET* inhibitors? Answers to these questions, along with the consensus recommendations herein, will help streamline the management of *MET*-altered NSCLC in routine practice, assist clinicians in therapeutic decision-making, and help ensure optimal outcomes for NSCLC patients with *MET* alterations.

## 1. Introduction

Although non-small-cell lung cancer (NSCLC) remains a leading cause of cancer-related death [1], the molecular characterization and classification of its genetic alterations and the subsequent development of targeted therapies has profoundly increased treatment options and overall survival (OS). The mesenchymal–epithelial transition (*MET*) proto-oncogene encodes the tyrosine kinase receptor for hepatocyte growth factor (HGF). Small molecule inhibitors of *MET* are recent additions to the NSCLC-targeted treatment armamentarium.

*MET* and its ligand, HGF, were characterized in the mid-1980s [2], and the first activating mutations identified within the *MET* gene were discovered by genome-wide analysis of families with hereditary papillary renal cell carcinoma [3]. Over the past two decades, alterations within and outside the *MET* kinase domain have been described in several solid tumours, including NSCLC, glioblastoma, breast, renal and colon cancers, as well as cancers of unknown primary origin, suggesting that activated *MET* plays a significant role in the tumourigenic process in a wide range of cell types [4,5,6,7,8,9,10,11].

*MET* is a single-pass transmembrane receptor composed of extracellular, transmembrane, juxtamembrane and tyrosine kinase domains as well as a carboxyterminal docking site [12]. The extracellular portion of *MET* is a binding site for HGF. Upon HGF binding, *MET* homodimerization results in the phosphorylation and activation of intracellular domains that stimulate several downstream signalling pathways, including the mitogen-activated protein kinase (MAPK)/extracellular signal-regulated kinase (ERK), phosphatidylinositol 3-kinase (PI3K)/protein kinase B (PKB), mammalian target of rapamycin (mTOR), and Janus kinase (JAK)/signal transducer and activator of transcription (STAT) pathways that promote cell migration, proliferation, and survival [13,14].

The *MET* gene, located on the long arm of human chromosome 7 (7q21–31), is approximately 125 kb in length and contains 21 exons [15]. Dysregulation of the *MET* pathway in lung cancer occurs through gene mutations, amplifications, fusions, rearrangements, and protein overexpression [16].

The most common *MET*-activating alterations in newly diagnosed non-squamous NSCLC are gene amplification (described in 2% to 5% of cases, depending on the different scoring systems adopted in clinical studies) and *MET* exon 14 (*MET*ex14) skipping mutations (occurring in 2% to 4% of cases) [17,18,19,20,21]. *MET*-activating alterations are also reported in 10–20% of patients with acquired resistance to epidermal growth factor receptor (EGFR) and anaplastic lymphoma kinase (ALK) inhibitors. [22,23] This indicates that *MET* alterations are sufficient to drive carcinogenesis both as a primary oncogenic driver and as a secondary driver of acquired resistance to targeted therapy [6,24]. Although it has been noted that *MET* overexpression can be detected by immunohistochemistry (IHC) in as many as 20–25% of patients [17], the correlation between *MET* IHC and *MET* genomic alterations is poor [25,26].

Several clinical trials have reported positive outcomes with *MET* inhibitors in three clinically relevant *MET* alterations:

1. *MET*ex14 skipping mutations
2. De novo *MET* amplification
3. *MET* amplification in acquired resistance to EGFR inhibitors

The trial results impact the management of NSCLC patients presenting with these three *MET* alterations and have created a need for consensus recommendations that can streamline and integrate diagnostic approaches to identify patients that can benefit from novel therapies. Moreover, due to the availability of other therapeutic options for these patients, including immune checkpoint inhibitors (ICIs), there is an added need for expert guidance on therapeutic approaches with optimal short- and long-term outcomes. Lastly, the therapeutic management of patients with advanced NSCLC and rare actionable mutations likely differs across Canada due to variations in access to molecular testing and drug funding, further highlighting the need for such recommendations.

## 2. *MET*ex14 Skipping Mutations

### Epidemiology, Clinical Features, and Prognostic Implications

*MET*ex14 skipping mutations are detected in 2–4% of lung adenocarcinoma cases [22,23,24], a prevalence comparable to *ALK*-rearranged lung cancer [27]. These mutations usually occur in older patients (median age of 72 years) with a higher percentage of ever-smokers compared to patients with tumours harbouring other oncogenic alterations such as *EGFR/ALK/ROS1* [21]. *MET*ex14 skipping mutations are usually mutually exclusive with other lung cancer-driving mutations, suggesting it is an independent oncogenic driver [19,20]. In a study of 933 patients with nonsquamous NSCLC, no patients with *MET*ex14 skipping mutations had activating mutations in *KRAS*, *EGFR* or *ERBB2*, or rearrangements involving *ALK*, *ROS1* or *RET* [20]. However, in another study that included 298 patients with *MET*ex14 skipping mutations, *KRAS* mutation was reported in 3% [21]. Co-occurring genomic alterations also include mutations in *TP53*, loss of *CDKN2A/B*, and amplification of *MET, MDM2*, and *CDK4/6* [21,22,23]. Furthermore, although a substantial proportion of *MET*ex14 lung cancers express PD-L1, the median tumour mutational burden (TMB) is lower compared with unselected NSCLCs [28].

A high frequency of *MET*ex14 skipping mutations has initially been reported in the NSCLC non- squamous subtype of pulmonary sarcomatoid carcinoma (PSC) [29], ranging from 5–32% of patients [30,31]. Most recently, the incidence of *MET*ex14 skipping mutations in patients with PSC was shown to be 7–8% [32,33]. *MET*ex14 skipping mutations have also been found in a very small percentage of patients with squamous cell carcinoma (SCC). Lam VK et al. reported *MET*ex14 alterations in 4 out of 385 (1%) SCC patients [34]. In two recent clinical trials that assessed the efficacy of *MET* inhibitors in patients with *MET*ex14 skipping mutations, GEOMETRY mono-1 [35] and VISION [36], patients with squamous and non-squamous histology were included. Squamous patients represented a significantly smaller proportion (8/97 patients in GEOMETRY-mono-1 and 7/99 patients in VISION with *MET*ex14 skipping mutations had SCC histology).

*MET*ex14 NSCLC is generally associated with aggressive disease, resistance to anticancer therapies, and poor prognosis when not treated with *MET* inhibitors [37,38,39,40]. Earlier studies indicate that the overall response rate (ORR) to ICIs in NSCLC patients with *MET*ex14 skipping mutations is low (approximately 16%), with a median PFS of approximately 2–5 months [28,41]. For first-line chemotherapy, a recent study revealed a median OS of about 9.5 months and a PFS of 4.0 months [42]

In a retrospective analysis of radiological features of NSCLC patients harbouring *MET*ex14 skipping mutations, the primary tumour seemed to present as solid and peripheral masses with a high frequency of multifocal and extrathoracic metastases, mainly to the bone, brain, and adrenal glands [43].

## 3. De novo *MET* Amplification

### Epidemiology, Clinical Features, and Prognostic Implications

*MET* amplification is thought to dysregulate *MET* pathway signalling via protein overexpression and constitutive kinase activation. De novo *MET* amplification occurs in approximately 1–5% (depending on the different scoring systems adopted in clinical studies) of NSCLC cases [6,44,45,46] and has been associated with poor survival in patients with surgically resected early-stage disease [26,44,47].

While *MET*ex14 skipping mutations are most commonly reported with adenocarcinomas given the overall incidence of this histology, *MET* amplification is more frequently identified in squamous cell carcinomas (SCC) [48]. Former or current smokers represent over 50% of patients with *MET* amplification in tumours, and a history of ever smoking is more common in SCC than in adenocarcinoma (71% vs. 34.3%; *p* < 0.001) [48]. The magnitude of *MET* amplification measured by *MET* gene copy number (GCN) status (≥5 copies/cell) does not appear to be associated with gender, smoking history, histology, or stage. Multivariable analysis showed that a higher *MET* GCN is significantly associated with shorter survival in SCC [48].

Patients presenting with *MET* amplification tend to be younger (average age of 66 years), male, and smokers. *MET* amplification occurs more frequently as a subclonal event, often co-occurring with other pathogenic mutations (including *NRAS, KRAS* and *TP53*), and is usually a late genetic event [16,49]. Co-occurrence of *MET* amplification in *MET*ex14 lung cancer is found in approximately 15–20% of cases, indicating that *MET* dependency in lung cancer can be driven by synergistic genomic events [21,38]. *MET* amplification is often present in other tumour types, including 6% of gastroesophageal carcinomas [50]. In both lung and gastric cancers, *MET* amplification is associated with higher histologic grade, advanced disease, and unfavourable prognosis [51].

## 4. *MET* Amplification as Acquired Resistance to EGFR Inhibitors

### Epidemiology, Clinical Features, and Prognostic Implications

*MET* amplification is a potential resistance pattern to EGFR inhibition in NSCLC, accounting for 5–22% of acquired resistance to first- and second-generation EGFR inhibitors [52,53,54]. In the phase III AURA3 trial, analysis of plasma samples from 73 *EGFR* T790M-positive patients with acquired resistance to the third-generation EGFR tyrosine kinase inhibitor (TKI) osimertinib showed that *MET* amplification is the most common resistance mechanism (19%), followed by *EGFR* C797S mutation (7%) [55]. Similar observations have been made in studies with rociletinib, another third-generation EGFR TKI that is no longer in development [56,57]. In the phase III FLAURA trial, next generation sequencing (NGS) analysis of 91 plasma samples from patients progressing on first-line osimertinib reported *MET* amplification in 15% of samples [58]. *MET* amplification is also detected in other oncogene-addicted NSCLCs including those with ALK rearrangements, in which 15% of tumour biopsies from patients relapsing on next-generation ALK inhibitors detected *MET* amplification [23].

The mechanism by which *MET* amplification causes resistance to EGFR inhibitors is associated with EGFR-independent phosphorylation of *ErbB3* and downstream activation of the PI3K/AKT pathway, bypassing EGFR inhibition [59]. Patients with *MET* amplification following osimertinib resistance tended to have inferior survival compared to patients without an increase in *MET* amplification (median PFS of 3.5 vs. 9.9 months; median OS of 15.6 months vs. 30.7 months) [60].

*MET* amplification after EGFR TKI may heterogeneously distribute the amount of metastatic sites; therefore, liquid biopsy may aid in detecting the alteration. A recent study showed that liquid biopsy could provide important insights into the heterogeneity of TKI resistance mechanisms in NSCLC [61].

## 5. Identifying Patients Most Likely to Benefit from *MET*-Targeted Therapies

### 5.1. Pre-Analytical Considerations

#### 5.1.1. Testing Strategies

Dysregulated *MET* expression and activity can be detected at the DNA, RNA, and protein levels. Assays commonly used in clinical trials include IHC to detect protein overexpression, fluorescence in situ hybridization (FISH) to identify gene amplification, reverse transcription polymerase chain reaction (RT-PCR) to detect gene mutations, and next-generation sequencing (NGS) to detect both amplifications and mutations, depending on the sample used (i.e., DNA and/or RNA).

Several studies demonstrate a poor correlation between IHC and *MET*ex14 skipping mutations and *MET* amplification, with negative predictive values of 95% for *MET* amplification and 94% for *MET*ex14 skipping mutations [25,26]. An analysis of the French IFCT-PREDICT.amm cohort, which consisted of 843 patients with treatment-naive advanced NSCLC, revealed similar rates of *MET*ex14 skipping mutations in patients with no or low vs. high *MET* expression by IHC [62]. Combined mutation and GCN analysis in patients with high *MET* protein-expressing tumours demonstrate that only 10% of these patients had *MET*ex14 skipping mutations or *MET* gene amplification. The majority of cases with high *MET* expression (*MET* 3+ immunoscore, i.e., ≥50% of tumour cells showing high-intensity staining) do not appear to be associated with alterations of the *MET* gene. Consequently, these findings do not support the use of *MET* IHC as a surrogate marker or screening for genomic *MET* alterations.

Laboratories aiming to implement clinical *MET* testing must select a methodology or testing algorithm that integrates all other standard-of-care lung cancer biomarkers reported within the recommended turnaround time. The methodology should take into consideration all other factors associated with the addition of the assays to the existing workflow (ease of use, technical hands-on time, and interpretation). The performance characteristics of the assays should be thoroughly validated with characterized samples representative of clinical practice, including small specimens. Other key considerations include the integration of complementary assays in order to respond to issues related to small specimens and assay failure/insufficiency rates, as well as cost-effectiveness.

Considering that patients with currently approved clinically actionable alterations (i.e., *EGFR, ALK, ROS1, RET* and *BRAF*) account for 15–20% of non-squamous NSCLC and rapid emergence of additional targets (e.g., *MET* alterations, *KRAS G12C*), a shift towards diagnostic platforms that allow multigene panel testing (e.g., NGS technology) is the most appropriate approach, as opposed to sequential single-gene testing such as FISH or RT-PCR. In this context, even if the relative number of patients with a specific molecular alteration represents only a small percentage of the whole NSCLC population, testing for new actionable drivers would have a minimal additional impact on service delivery. If sufficient numbers of mutations are included, upfront NGS represents a feasible, cost-effective method of diagnostic molecular profiling compared with sequential testing strategies [63]. Furthermore, having in-depth knowledge of the mutation status of patients is critical as patients with some oncogenic drivers might have an inferior clinical benefit from alternative therapies such as ICIs. Patient identification is essential for choosing an individualized therapeutic strategy [64].

Multiplex gene testing is supported worldwide by several international pathology and oncology association guidelines [65,66,67]. In addition, the majority of European countries have adopted this approach and developed country-specific recommendations [68].

Testing for *MET*ex14 skipping mutations should be performed for all treatment-eligible patients with advanced non-squamous NSCLC, irrespective of clinical characteristics such as patients with PSC, due to the higher frequency of *MET*ex14 skipping mutations found in these tumours. Ideally, reflex testing should be initiated routinely by the pathologist at the diagnosis stage in patients with advanced-stage non-squamous NSCLC. The advantages of reflex testing include optimal tissue management and reduced waiting time for results [69,70,71]. In patients with SCC, testing should be performed at a minimum in non-smokers with advanced disease that are treatment-eligible, on-demand by a clinician. Based on access to local testing, all patients with advanced SCC irrespective of smoking status could be tested, given the presence of *MET* alterations in this patient population. This would improve biomarker testing in this group and increase the number of patients with SCC that would be able to access targeted therapy if appropriate.

#### 5.1.2. Tissue versus Liquid Biopsy

Histology is currently the gold standard for molecular analysis in NSCLC. However, as a large proportion of NSCLC patients present with advanced disease and associated health-related risks, tissue samples for molecular testing are predominantly small biopsies and cytological samples. In certain situations (e.g., tissue biopsy with scarce tumour cells; time for tissue biopsy is too lengthy; invasive procedures are contraindicated), liquid biopsy could be considered where available.

Numerous studies have demonstrated a relatively good correlation between liquid and tissue biopsy results and the potential of liquid biopsy with cell-free DNA, including circulating tumour DNA (ctDNA), to detect actionable genomic alterations including *MET* alterations [72,73]. Thus, driver mutations found by liquid biopsy should be considered actionable.

Circulating tumour DNA, a subset of total cell-free DNA, is released through lysis of apoptotic and necrotic cells, digestion of tumour cells by macrophages, or by direct secretion of DNA by tumour cells [74]. The fraction of ctDNA varies depending on tumour stage, disease burden, vascularization, and biological features (i.e., apoptotic rate, metastatic potential of the cancer cells) [75,76]. The half-life of ctDNA in the bloodstream varies between 16–150 min, making ctDNA a “real-time” biomarker that reflects tumour burden [74].

When selecting a liquid NGS assay, one should consider the coverage of *MET* introns, the ability to detect copy number alterations, and the differences between approaches using ctDNA and those using both ctDNA and ctRNA (see analytical considerations for more detail). On top of this, the overall limitations of plasma-based NGS, including sensitivity as it relates to the stage and burden of disease, should be considered.

#### 5.1.3. Turnaround Time and Reporting of Biomarker Test Results

Rapid turnaround time for biomarker test results, especially in newly diagnosed NSCLC requiring therapy, is extremely important for timely treatment initiation. The College of American Pathologists recommends a maximum 10-day turnaround time from sample receipt in the laboratory to report generation [65]. It is also recommended that the pre-laboratory turnaround time does not exceed three business days and that the post-laboratory turnaround time is less than 24 h [77]. However, the maximum acceptable time to wait for biomarker results for each patient should be at the clinician’s discretion and balanced between missed treatment opportunities and the benefits of waiting for appropriate targeted therapy. The biomarker test results should be compiled and ideally reported in a single comprehensive report by the pathologist, including PDL1 status. This is the optimal process to guide treatment decisions, as PDL1 status alone for patients with non-squamous histology is insufficient to guide treatment decisions.

#### 5.1.4. Recommendations

1. Testing for *MET*ex14 should be performed as part of a comprehensive panel that includes current standard-of-care biomarkers as summarized by international guidelines. All advanced stage non-squamous NSCLC patients, including patients with PSC and those without alterations in *EGFR*, *ALK* or *ROS1* should be tested, regardless of clinicopathologic characteristics.
2. Reflex biomarker testing for *MET*ex14 skipping mutations should be initiated by the pathologist at the time of initial diagnosis in all patients diagnosed with advanced-stage non-squamous NSCLC.
3. *MET*ex14 testing in advanced SCC should be performed upon the oncologist’s request in treatment-eligible non-smokers.
4. Considering the availability of multigene panels, there is increasing consensus that smokers with advanced SCC that are treatment-eligible should also be considered for testing given the presence of *MET* alterations in this population.
5. Liquid biopsy should be considered if a tissue biopsy is unavailable, inadequate for molecular testing, when invasive procedures for tissue procurement are contraindicated, or when urgent treatment decisions are required and delays are expected with tissue testing.
6. Negative results by liquid biopsy do not mean the absence of the target; if possible, reflex to tissue testing is recommended.
7. Regardless of the type of biopsy (tissue or liquid), identified actionable genomic alterations, including *MET*ex14, are acceptable as valid indications for approved *MET* targeted treatments.
8. The maximum acceptable turnaround time (from the acquisition of tissue to the oncologist having the report) for all biomarkers should not exceed 21 calendar days. In certain situations, accelerated testing should be available.
9. Biomarker test results should be compiled and ideally reported in a single comprehensive biomarker report by the pathologist, including PD-L1 expression.

### 5.2. Analytical Considerations

#### 5.2.1. Detection of *MET*ex14 Skipping Mutations

*MET*ex14 skipping mutations are a heterogeneous group of indels and missense mutations that result in post-translational modifications detectable at the RNA level, which poses challenges and requires specific analytical and diagnostic considerations [78]. Genomic characterization of *MET*ex14 samples has shown that DNA changes display remarkably diverse sequences, with variants extending deep into intronic non-coding regions adjacent to exon 14 [19]. Genomic variants altering or ablating a splicing site must be detected when using a DNA-based approach, while RNA sequencing allows characterization of altered splicing and fusion of exon 13 to 15 regardless of underlying genomic alteration. For clinical use in NSCLC where comprehensive biomarker testing is warranted, the main types of strategies offered by commercial assays include amplicon-based and hybrid-capture-based NGS. Several studies have explored their performance in comparison to conventional molecular assays (i.e., RT-PCR, Sanger sequencing) [78,79,80].

The main limitation of small DNA-based amplicon panels is improper coverage. Comparison of such panels with RNA-based anchored-multiplex (AMP)-PCR revealed a higher incidence of identified alterations in NSCLC samples tested by the RNA-based assay (4.2% versus 1.3%), with 6 of 10 AMP-PCR-positive results negative on the DNA-based assay [80]. This is in line with an in silico study which reported that commercially available DNA-based NGS panels could only detect 63% of literature-described splicing mutations associated with *MET*ex14 [81]. However, performance was improved after the customization of panels with additional *MET* amplicons [82]. Nonetheless, the interpretation of an *MET*ex14 variant without confirmatory splicing alteration with RNA sequencing can be problematic in some circumstances.

The role of complementary RNA sequencing for proper identification of *MET*ex14 also applies for hybrid-capture panels. This was shown in 252 driver negative NSCLC samples, based on a large DNA-based NGS panel that underwent AMP-PCR RNA sequencing [83]. Six (2.5%) *MET*ex14-positive cases were revealed amongst other oncogenic fusions, of which five cases had intronic variants located as far as 40 bp away from the intron 13 splice site on manual review of the DNA sequencing data [83]. Considerations involved in such false-negative cases, outside of assay design with improper genomic coverage of regions involved in splicing (large intronic regions as well as branch site, polypyrimidine tract, splice acceptor and donor site of *MET* exon 14), include large genomic deletions to primer binding sequences as well as bioinformatics filtering [80,83]. On the other hand, RNA-based assays are highly dependent on RNA quality [80]. All of these considerations, which affect FFPE- and plasma-based NGS, highlight the need for properly validated assays and complementary techniques in some clinical contexts (e.g., driver negative case) in order to achieve optimal *MET*ex14 detection.

#### 5.2.2. Recommendations

10. *MET*ex14 testing methodology should undergo specific validation of performance characteristics before clinical implementation, whether it is based on a hybrid-capture- or amplicon-based NGS strategy on DNA or in combination with or complemented by RNA-based NGS.
11. Due to a high risk of poor sensitivity, caution is needed when amplicon-based DNA panels are used to capture some genomic *MET*ex14 skipping mutations without combined RNA sequencing.
12. Clinicians and pathologists should be aware of and consider assay limitations when interpreting results, including whether a particular assay includes intronic regions of *MET* and whether it is capable of identifying skipping alterations.
13. Although the panel does not recommend single-gene testing for *MET*ex14 skipping mutations, additional multi-target testing is recommended for patients who are driver-unknown following single-gene testing. The selection of a proper molecular method should follow the same considerations as for NGS in terms of performance.
14. *MET* IHC is not recommended as a screening tool for *MET*ex14 skipping mutations, as the data indicate a poor correlation between *MET* IHC and *MET*ex14 skipping mutations.

#### 5.2.3. *MET* Amplification

*MET* amplification is thought to dysregulate *MET* pathway signalling via protein overexpression and constitutive kinase activation; however, it is unclear whether *MET* amplification levels change over time and/or after some therapies. *MET GCN* is a continuous variable, and the definition of a positive threshold affects incidence, rate of overlap with other genotypes, and ability to predict the efficacy of *MET* inhibitors [84].

The most frequent technologies used to assess *MET GCN* variations in the clinical setting are FISH and NGS. While FISH is conventionally used to assess amplification, NGS is becoming routine in molecular diagnostics and provides a means to assess *MET* amplification in the context of comprehensive genomic profiling.

Although *MET* inhibitor response rate is largely driven by copy number, a consensus on the definition of *MET* positivity based on GCN has yet to be reached. With FISH, two different quantification criteria are used: either an increase in absolute copy number (e.g., mean copy number of the gene per cell), or an increase in the ratio of gene copies relative to other areas on the same chromosome (typically the centromere of the chromosome where the gene is located, that is, the ratio of *MET* to chromosome 7 centromere, *MET/CEP7*) [84].

Recent clinical trials with *MET* inhibitors define different cut-offs for *MET* amplification positivity. The PROFILE 1001 with crizotinib used *MET*/*CEP7* ratios and defined cut-offs of ≥1.8 to ≤2.2 as low, >2.2 to <4 as medium, and ≥4 as high *MET* amplification [85]. The cut-offs assessed in the GEOMETRY-mono 1 trial with capmatinib were GCNs of <4, 4 to 5, 6–9 and >10 [35]. Finally, the most recent analysis from the VISION trial with tepotinib, which utilized liquid biopsy, defined *MET* amplification positivity as *MET* GCN > 2.5 [86].

#### 5.2.4. Recommendations

15. When selecting an NGS panel for use in NSCLC, pathologists are encouraged to utilize an assay that provides copy number status, and to ensure that copy number coverage of *MET* is included.
16. When reporting the *MET* copy number status or copy number ratio, it is important that pathologists and end-users are aware of the particular cutpoints being used. While no definitive cutpoints have been established, practitioners are encouraged to monitor the emerging data on this topic.
17. Users should be aware of any limitations to copy number assessment by NGS, such as low uniformity and low tumour content.
18. In select scenarios, single-gene tests for *MET* amplification, such as FISH, can be utilized. This could include patients with resistance to EGFR TKI therapy.
19. *MET* IHC is not recommended as a screening tool for *MET* amplification, as the data indicate a poor correlation between *MET* IHC and *MET* amplification.
20. Liquid biopsy may be considered, particularly when testing for *MET* amplification as a resistance mechanism, while recognizing the lower sensitivity of plasma-based assays compared to tumour tissue testing. The limitations of liquid NGS in general, as well as the particular assay, should be considered. However, *MET* amplification detected by plasma-based assays should be considered actionable.

## 6. What Are the Preferred First-Line and Subsequent Therapies for Patients with Advanced NSCLC Harbouring *MET*ex14 Skipping Mutations?

- What is the preferred first-line therapy for treatment-naïve patients with *MET*ex14 skipping mutations?
- What are the preferred subsequent lines of therapy for patients with *MET*ex14 skipping mutations unexposed to *MET* inhibitors?

### 6.1. ICIs in Patients with METex14 Skipping Mutations

The results of the KEYNOTE 024 [87], KEYNOTE 189 [88] and KEYNOTE 407 [89] trials demonstrate favourable long-term outcome effects with ICI monotherapy in patients with a tumour proportion score for programmed death-ligand 1 (PD-L1) of ≥50%, as well as with an ICI in combination with chemotherapy. In the Checkmate 227 trial, first-line nivolumab plus ipilimumab improved OS compared to chemotherapy in patients with NSCLC, independent of PD-L1 expression level [90]. Next, the Checkmate 9LA trial confirmed that nivolumab plus ipilimumab with two cycles of chemotherapy provided a significant improvement in OS versus chemotherapy alone [91]. All of these trials excluded patients with *EGFR* or *ALK* mutations, but not those with *MET*ex14 skipping mutations. However, the number of patients included with these alterations in these studies has not been reported. Based on these trials, recent recommendations from the American Society of Clinical Oncology (ASCO) and Ontario Health (OH; Cancer Care Ontario) consider ICI ± chemotherapy as the standard of care for treatment-naïve *EGFR* and *ALK*-negative stage IV NSCLC [92].

A large international retrospective study (IMMUNOTARGET) assessed the benefits of ICIs in 551 patients with advanced NSCLC and oncogenic driver alterations [41]. The study included 36 patients with *MET* alterations (76.5% former or current smokers; 23 with *MET*ex14, and 13 with *MET* amplification). There was a trend toward longer median PFS with an ICI in patients with *MET*ex14 alterations (4.7 months) compared to those with *MET* amplification (1.3 months, *p* = 0.09). Median PFS did not correlate with smoking status. Median OS was 25 months in patients with *MET*ex14 skipping mutations and 8.0 months in those with *MET* amplification. The objective response rate (ORR) in the entire *MET* cohort was 16%.

Similar responses were achieved by Sabari et al. [28]. Among 111 evaluable tumour samples from NSCLC patients with *MET*ex14 skipping mutations, a PD-L1 expression of 0%, 1–49%, and ≥50% was seen in 37%, 22%, and 41% of samples, respectively. The median tumour mutational burden (TMB) of tumours harbouring *MET*ex14 skipping mutations was lower than that of unselected NSCLCs, and there was no association between PD-L1 expression and TMB. Of the 111 cases, only 24 were response-evaluable, in whom the ORR was 17% and the median progression-free survival (PFS) was 1.9 months. Response was not enriched in tumours with PD-L1 expression ≥ 50% or high TMB. The authors concluded that occasional response to PD-1 blockade could be achieved; however, overall clinical efficacy was modest. In contrast to these data, some recently published small case series have reported response in about 50% of cases and PFS of over two years in patients treated with ICIs [93,94,95].

A report on over 1300 NSCLC patients with *MET*ex14 skipping mutations confirmed that TMB is significantly lower in these patients compared to *MET*ex14 wildtype NSCLC (3.6 vs. 7.0 mut/Mb) [96]. *MET*ex14 NSCLC was also enriched for high (≥50%) PD-L1 positivity compared to wildtype NSCLC (48% vs. 29%). The report, however, did not include other relevant patient characteristics (i.e., smoking status), and the authors concluded that additional data are needed to determine the predictive role of these biomarkers for ICI response in patients with NSCLC harbouring *MET*ex14 skipping mutations. Several studies reported that PD-L1 expression in *MET*ex14 tumours could be relatively high (≥50% expression in 40–70%) [28,96,97]. This raises questions about the relevance of PD-L1 expression as a biomarker for patients with advanced *MET*ex14 NSCLC, as well as the safety and efficacy of ICIs in this population; notably the balance between potential efficacy and toxicity associated with receiving TKIs, in particular crizotinib, following ICI therapy [95,98]. According to Canadian real-world data presented at ASCO 2021, six out of seven patients receiving crizotinib post-ICIs experienced an early grade ≥ 3 AE (four transaminitis, two pneumonitis) resulting in permanent discontinuation of TKI in half of the patients [95]. Biomarkers and characteristics that could indicate which patients will have ICI-related events are unknown.

Based on clinical trial data and current practice, it is apparent that the optimal sequence of therapy in patients with *MET*ex14 NSCLC is yet to be determined. Several factors are involved in therapeutic decision-making, including the severity of disease, smoking status (which could impact TMB), likelihood of response to ICIs, and the likelihood of rapid deterioration. Based on the limited evidence, there are some concerns that, similar to activity in other driver mutations (EGFR and ALK), monotherapy ICIs may not have robust activity in patients with *MET*ex14 skipping mutations. However, unlike patients with *EGFR* and *ALK,* where a majority of patients are non-smokers, 50% of patients with *MET*ex14 alterations present with a history of smoking. Patients with PSC tend to be very heavy smokers, and often respond well to ICIs, especially if there is high PD-L1 expression [99,100], whereas non-smokers have less benefit from ICIs [101]. Thus, the panel recognized that smoking status may be implicated in decision-making and optimal sequencing of therapy in NSCLC patients with *MET*ex14 skipping mutations.

### 6.2. MET Inhibitors in Patients with Advanced METex14 Skipping Mutations

Multiple *MET* inhibitors, including both small molecule TKIs and monoclonal antibodies against *MET* or HGF have been in clinical development since the early 2000s [102]. *MET* TKIs can be divided into type I and type II, based on their binding site (Table 1) [103], and both types are ATP-competitive. Type I binds to *MET*’s unique autoinhibitory conformation by interacting with Y1230 in the *MET* activation loop, while type II binds to the adenosine binding site and extends to the hydrophobic back pocket.

Type I inhibitors can be further divided into type Ia (crizotinib) and type Ib (Table 1). Type Ib inhibitors are highly specific for *MET* and have fewer off-target effects compared to type Ia.

#### 6.2.1. Crizotinib

Crizotinib is a multikinase inhibitor with strong activity against *ALK, ROS1* and *MET* alterations. The antitumour activity and safety of crizotinib (250 mg twice daily) was assessed in the PROFILE-1001 trial, which included 69 patients with advanced NSCLCs harbouring *MET*ex14 skipping mutations [85]. ORR was 32% among 65 response-evaluable patients and there was no difference in ORR by type of *MET* alteration, either by splice-site region (32% for splice donor site, 31% for splice acceptor site) or by mutation type (36% for base substitution, 25% for indel). The median DOR was 9.1 months. Median PFS was 7.3 months and 54% of participants were progression-free at 6 months. Median OS was estimated at 20.5 months, with 6- and 12-month rates of 87% and 70%, respectively. Elevated transaminase levels and dyspnea were the most frequent grade 3 treatment-related adverse events (TRAEs), with each observed in 4% of participants. There was one case each of grade 4 hypophosphatemia, lymphopenia, pulmonary embolism, and one fatal case of treatment-related interstitial lung disease.

The antitumour activity of crizotinib was also confirmed in a phase II prospective multicentre two-arm trial in patients with NSCLC with *ROS1* rearrangements (cohort A; *n* = 26) or *MET* deregulation (cohort B; *n* = 26) [104]. Cohort B included nine patients with *MET*ex14 skipping mutations, sixteen with *MET* amplification (threshold *MET*/*CEP7* > 2.2), and one with both amplifications and *MET*ex14 skipping mutations. In the entire *MET* cohort, eleven patients (42%) had stable disease (SD), for an overall disease control rate (DCR) of 69%. With a median follow-up of 21 months, median PFS, median OS and DOR were 4.4 months, 5.4 months, and 3.7 months, respectively. Out of nine patients with *MET*ex14 skipping mutations, only one had a partial response, although five had SD (DCR 67%).

These trials indicate that although crizotinib has some activity in patients with *MET* alterations, the ORR with crizotinib in these patients is lower compared to that achieved with targeted therapy for many other NSCLC driver mutations. Although crizotinib was the first *MET* TKI to be evaluated in this patient group, more promising data have been reported with more selective agents.

#### 6.2.2. Tepotinib

The phase II VISION trial assessed the efficacy of tepotinib (500 mg once daily), a type 1b *MET* inhibitor, in patients with *MET*ex14 skipping mutations (cohort A) [36]. In treatment-naïve patients (*n* = 65), ORR was 44.6%, median DOR was 10.8 months (Table 2), and PFS was 8.5 months. In previously-treated patients (*n* = 81), ORR was 45.7%, median DOR was 11.1 months, and median PFS was 10.9 months. Tepotinib was generally well tolerated across therapy lines, with mostly mild to moderate AEs and few discontinuations. The most common TRAE, peripheral edema (63% all grades; grade ≥ 3, 7.5%), rarely led to discontinuation (4%). Other common AEs included nausea (26%), diarrhea (22%), creatinine increase (18%), and hypoalbuminemia (16%).

Based on this data, Health Canada has issued a marketing authorization for tepotinib with conditions, pending the results of trials to verify its clinical benefit. In Canada, tepotinib is indicated for the treatment of adult patients with locally advanced unresectable or metastatic NSCLC harbouring *MET*ex14 skipping alterations.

#### 6.2.3. Capmatinib

In the GEOMETRY mono-1 trial, a multicentre, open-label phase II study evaluated the efficacy and safety of capmatinib monotherapy in adult patients with *EGFR* wildtype, *ALK*-negative rearrangement, advanced NSCLC harbouring *MET*ex14 skipping mutations and/or *MET* amplification [35]. Patients with *MET*ex14 skipping mutations were assigned to cohorts 4 (previously treated patients) or 5b/7 (treatment-naïve) and received 400 mg capmatinib twice daily. The primary endpoint was ORR based on blinded independent review committee (BIRC) assessment per Response Evaluation Criteria in Solid Tumors (RECIST v1.1). The key secondary endpoint was DOR by BIRC. ORR in treatment-naïve patients (*n* = 60) was 67.9% for cohort 5b and 65.6% for cohort 7; the combined ORR for treatment naïve patients was 66.7%. Median DOR was 12.6 months for cohort 5b (Table 2). Median DOR, PFS and OS for cohort 7 have not yet been reached. ORR in the previously treated patients (*n* = 69) was 40.6% and median DOR was 9.7 months. A clinically meaningful median OS of 20.8 months in first-line (Cohort 5b) and of 13.6 months in relapse settings (Cohort 4) was also observed [105].

Across all cohorts (364 patients), the most commonly reported AEs, regardless of causality, were peripheral edema (51%; 9% grade 3 or 4), nausea (45%), vomiting (28%), and increased creatinine (24%; 0% grade 3 or 4) [35]. Grade 3/4 TRAEs, regardless of causality, were reported in 67% of patients.

The U.S. Food and Drug Administration (FDA) granted accelerated approval to capmatinib for adult patients with metastatic *MET*ex14 NSCLC detected by an FDA-approved test.

#### 6.2.4. Savolitinib

The efficacy and safety of savolitinib 600 mg (bodyweight ≥ 50 kg) or 400 mg (bodyweight < 50 kg) in unresectable or metastatic *MET*ex14 NSCLCs (40 (57%) with adenocarcinoma, 25 (36%) with PSC and 5 (7%) with other NSCLC subtypes) were assessed in a multicentre, multi-cohort, single-arm phase II study [106]. At a median follow-up of 17.6 months, the IRC-assessed objective response rate was 42.9% (30 of 70 patients). The median time to response was 1.4 months, the median DOR was 8.3 months, and the disease control rate was 82.9% (72% in PSC and 88.9% in other types of NSCLC). Seven (10%) patients had a DOR that lasted 12 months or more. The median PFS was 6.8 months, and the 6-month and 12-month PFS was 52.0% and 32%, respectively. All 70 patients reported at least one TRAE. TRAEs of grade ≥ 3 occurred in 32 (46%) patients, the most frequent of which were increased aspartate aminotransferase (*n* = 13%), increased alanine aminotransferase (10%), and peripheral edema (*n* = 9%). Treatment-related serious adverse events occurred in 17 (24%) patients, the most common being abnormal hepatic function (4%) and hypersensitivity (2.9%).

Savolitinib has been granted conditional approval in China to treat patients with NSCLC with *MET*ex14 skipping alterations who have progressed following prior systemic therapy or are unable to receive chemotherapy.

In some countries, *MET* inhibitors are approved along with a companion diagnostic (CDx). The U.S. FDA has approved FoundationOne^®^ Liquid CDx for use with capmatinib [107]. ArcherMET CDx was approved by the Japanese Ministry of Health to detect *MET*ex14 skipping alterations in tissue (RNA) and liquid biopsy for tepotinib. The U.S. FDA, however, has not specified a CDx for selecting eligible patients for tepotinib [108]. As current data indicate the efficacy of *MET* inhibitors as both first- and second-line therapy, the first-line choice should be based on patient characteristics (i.e., smoking status, comorbidities, and contraindications), disease characteristics (i.e., PD-L1 expression), as well as disease severity and the likelihood of fast rapid deterioration. In the Canadian context, one must also consider access to treatment and the balance between missed treatment opportunities while waiting to access *MET*-targeted therapy.

### 6.3. Recommendations

21. Patients with advanced *MET*ex14 NSCLC (non-squamous and squamous histology) that are eligible for treatment should be offered *MET* targeted therapy at some point during the course of their disease. Caution is needed when initiating a TKI post-ICI due to the potential increased risk of toxicity.
22. Although crizotinib has shown efficacy in patients with *MET*ex14 skipping mutations, regulatory approval for this indication was not sought. Due to more robust efficacy, type 1b *MET* inhibitors (tepotinib, capmatinib, or savolitinib) are preferred over crizotinib; however, crizotinib is listed on some provincial formularies and may be considered as an option if other therapies are unavailable.
23. The choice between type 1b *MET* inhibitors should be based on patient preference, toxicity profile, regulatory approvals, and access.
24. Patients with advanced *MET*ex14 NSCLC may be offered first line therapy with *MET*-targeted therapy or other guideline-recommended standard of care approaches for patients without driver mutations. Based on current data with ICI in non-smokers and the evidence with *MET* inhibitors, an *MET* inhibitor is the preferred first-line therapy in non-smokers with NSCLC and *MET*ex14 skipping mutations.
25. For patients exposed to *MET*-targeted therapy, the guideline-recommended standard of care for advanced NSCLC patients without driver mutations should be offered as a subsequent line of treatment.

### 6.4. What Are the Treatment Options for Metastatic NSCLC Patients with METex14 Skipping Mutations and Brain Metastases?

Brain metastases may develop in up to 20–40% of patients with stage IV NSCLC [109], and the incidence among NSCLC patients with *MET*ex14 skipping mutations is similar [110]. Activity of therapy against brain metastases is essential for maintaining the best disease response and quality of life.

An open-label, phase II study indicated the efficacy of ICIs in PD-L1 positive (PD-L1 expression ≥1%) stage IV NSCLC with brain metastasis, with 29.7% of patients achieving a response [111]. 93% of patients were former or current smokers and 53% had driver mutations (fourteen KRAS, six EGFR, and one each ALK, HER2, and *MET*ex14).

The efficacy of crizotinib in patients with brain metastases is limited due to poor brain penetration; in humans, a low CSF-to-plasma ratio (0.26 to 1) has been reported [112].

In the GEOMETRY mono-1 trial, fourteen patients with *MET*ex14 skipping mutations had brain metastases [35]. Intracranial responses to capmatinib were observed in seven of thirteen (54%) evaluable patients, including complete resolution in four patients. Three of the seven patients with a response had received previous radiotherapy that could have contributed to response.

In the VISION trial (Cohort A), 23 patients had brain metastases at baseline, 15 of which were evaluable by the Response Assessment in Neuro-Oncology Brain Metastases (RANO-BM) criteria [113]. Twelve patients had received prior radiotherapy (2.6 to 44 weeks before study entry). Out of the seven patients with measurable disease, the intracranial best overall response (BOR) to tepotinib was 71% (three of the five patients that responded had complete disappearance of the target lesions). Of eight patients with non-target lesions only, seven achieved intracranial disease control, and one had PD [113]. Moreover, case reports suggest a prolonged intracranial response to tepotinib in patients with symptomatic brain metastases from lung adenocarcinoma and *MET* alterations [114,115,116].

Data with capmatinib and tepotinib indicate that selective type Ib *MET* inhibitors, which include tepotinib and capmatinib, are valid therapeutic options for NSCLC patients with brain metastases harbouring *MET*ex14 skipping mutations. As data is limited in determining the efficacy of *MET* inhibitors for treatment of brain metastases versus local therapies with radiation or surgery, these patients require multidisciplinary discussions to determine their optimal care.

### 6.5. Recommendations

26. For NSCLC patients with brain metastases harbouring *MET*ex14 skipping mutations, the type Ib *MET* inhibitors tepotinib and capmatinib may be considered as reasonable therapeutic options in addition to other multidisciplinary approaches. Such cases should be discussed with a multidisciplinary team including but not limited to radiation oncologists, neurosurgeons and medical oncologists.

## 7. What Are the Preferred First-Line and Subsequent Therapies for Advanced NSCLC Patients with De Novo *MET* Amplification?

The prevalence of high *MET* GCN gain and amplification carries a poor prognosis. Yet, due to challenges associated with the detection and reporting of *MET* amplification in NSCLC, studies assessing the efficacy of different therapeutic approaches in patients with de novo *MET* amplifications are sparse. About 15% to 20% of patients with *MET*ex14 skipping mutations will also have an *MET* amplification [39,49]. This section will focus on patients without a concurrent *MET*ex14 skipping mutation. Current data indicate the impact of GCN on response to *MET* inhibitors (Table 3).

In a small series, the ORR with crizotinib differed dramatically between cases with different *MET/CEP7* ratios (for a ratio of 1.8 to ≤2.2, ORR = 0%; for a ratio of >2.2 to <5, ORR = 17%; and for a ratio of ≥5, ORR = 67%) [117]. One should keep in mind that *MET/CEP7* ≥ 5 represented only 0.34% of adenocarcinomas [82]. Out of seventeen patients with *MET* amplifications in the Cohort B of the METROS study (threshold *MET/CEP7* > 2.2), six (35%) achieved PR and six (35%) SD with crizotinib [104].

In PROFILE 1001, the ORR in patients with high *MET* amplification category (≥4 *MET/CEP7* ratio) treated with crizotinib was 38.1%, compared to 14% in patients with medium amplification category (>2.2 to <4 *MET/CEP7* ratio) [118]. Median DOR and median PFS in high *MET* amplification patients was 5.2 and 6.6 months, respectively, compared to a DOR of 3.8 and PFS of 1.9 months in patients in the medium category. *MET* amplification GCN ≥ 6 was detected by NGS in fifteen of nineteen (78.9%) patients. Of these, ORR was observed in six (40%), two of whom had concurrent *MET*ex14 skipping mutations. No responses were observed among five patients with concurrent *KRAS*, *BRAF*, or *EGFR* mutations.

In GEOMETRY mono-1, cohorts with *MET* FISH GCN ≤ 9 (Cohorts 1b, 2 and 3) were closed for futility (overall response 7–12% and OS 2.7–3.6 months with capmatinib) at the interim analysis [35]. Capmatinib showed activity in patients with GCN ≥ 10. ORR as assessed by the independent review committee was observed in 29% of 69 previously treated patients and in 40% of 15 patients who had not received previous treatment; however, the results were lower than the prespecified threshold for significance. Nevertheless, capmatinib was assessed by the independent review committee to have clinically relevant efficacy if a response was observed in at least 35% of the patients, with a lower boundary of the 95% confidence interval of more than 25%. Median DOR was 8.3 months among twenty previously treated patients and 7.5 months among six patients who were treatment naive; the median PFS was 4.1 months and 4.2 months, respectively.

Cohort B of the VISION trial is currently assessing the efficacy of tepotinib in patients with *MET* amplification. The liquid biopsy analysis will include all subjects who tested positive for *MET* amplification in plasma ctDNA (GCN ≥ 2.5), irrespective of the tissue biopsy result. Two additional tissue biopsy analysis sets may be explored irrespective of the liquid biopsy test result: first, subjects with GCN gain ≥ 4 and <6, and second, subjects with a GCN gain of ≥6. The first analysis presented at ASCO 2021 included 24 patients with *MET* amplification detected by Guardant360 liquid biopsy assay with *MET* GCN ≥ 2.5. Response rates in the seven patients that received tepotinib as first-line therapy was 42%, and 30% in the ten patients that were treated using it as second-line [86].

Although preliminary evidence indicates activity of *MET* inhibitors in patients with NSCLC and *MET* amplification, the number of evaluated patients in these trials is small, duration of the follow-up is short, and the amplification thresholds are not clearly defined and vary between the trials. On the other hand, current standard of care approaches for patients without actionable mutations (ICIs ± chemotherapy) lead to a median PFS of 8–9 months and a median OS of 16–30 months [91,119,120,121,122]. After discussing evolving data with *MET* inhibitors and evidence in support of ICIs ± chemotherapy, the panel concluded that ICIs ± chemotherapy should remain the standard of care in NSCLC with de novo *MET* amplification.

### Recommendations

27. In patients with advanced NSCLC with de novo *MET* amplification, *MET*-targeted therapy could be considered through clinical trials at any line of therapy.
28. In patients with advanced NSCLC with de novo *MET* amplification, *MET*-targeted therapy could be considered after other standard therapies have been exhausted or in cases not eligible for standard therapies.

## 8. What Is the Preferred Therapy for Patients with Advanced EGFR-Mutated NSCLC with Acquired *MET* Amplification Progressing on EGFR Inhibitors?

*MET* amplification is a potential resistance pattern to first- and second-generation EGFR inhibitors in NSCLC [50,51,52]. *MET* amplification is also recognized as a resistance mechanism to osimertinib in first- and subsequent-line therapy in patients with a T790M mutation. In the FLAURA trial, which evaluated first-line osimertinib in EGFR-mutated NSCLC patients [58], and the AURA3 trial, which evaluated osimertinib in patients with acquired T790M resistance [123], paired plasma samples were collected at baseline and at progression. *MET* amplification was detected in 15% of the paired samples following first-line osimertinib, and 19% following progression on osimertinib in EGFR T790M positive patients.

As patients with *MET* amplification following osimertinib resistance tended to have inferior outcomes compared to patients without an increase in *MET* amplification [58], there is a need for effective strategies for patients who develop acquired resistance to osimertinib due to *MET* amplification. As *MET* amplification causes resistance to EGFR inhibitors by activation of an EGFR-independent pathway [58], targeting both EGFR and *MET* is required in order to overcome resistance to EGFR inhibitors.

A phase Ib/II trial assessed the efficacy of capmatinib plus gefitinib in patients with EGFR-mutated, *MET*-dysregulated NSCLC who experienced disease progression while receiving first-generation EGFR inhibitors (gefitinib, *n* = 72; erlotinib, *n* = 30; and afatinib, *n* = 4) [124]. The trial reported an ORR of 27%, while a 47% ORR was reported among patients with high *MET* amplification (≥6 GCN).

The phase Ib/II INSIGHT trial [125] that compared tepotinib plus gefitinib versus tepotinib plus chemotherapy in patients with *MET* overexpression (high IHC3+ or medium IHC2+) or *MET* amplification with acquired resistance to EGFR inhibition (the majority of patients were exposed to first- or second-generation EGFR inhibitors and only 2 out of 73 received prior osimertinib), also reported benefit from dual *MET* and EGFR inhibition (mean GCN ≥ 5 or *MET/CEP7* ratio ≥ 2). PFS and OS were longer with tepotinib plus gefitinib than with chemotherapy in patients with high (IHC3+) *MET* overexpression (*n* = 34; median PFS 8.3 months vs. 4.4 months; HR 0.35; median OS 37.3 months vs. 17.9 months; HR 0.33) or *MET* amplification (*n* = 19; median PFS 16.6 months versus 4.2 months; HR 0.13; median OS 37.3 months versus 13.1 months; HR 0.08). Although the trial was terminated early due to poor recruitment, these findings suggest improved anti-tumour activity for tepotinib plus gefitinib compared to standard chemotherapy in patients with EGFR-mutant NSCLC and *MET* amplification. The investigators also suggested that the use of liquid biopsy for detection of *MET* amplification instead of tissue biopsy could be a convenient and less invasive method that might improve trial recruitment.

In the phase Ib TATTON trial, 64% of patients with EGFR mutation-positive NSCLC and *MET*-amplified tumours (*MET/CEP7* ratio ≥ 2 or mean GCN ≥ 5) progressing on first-generation EGFR TKI responded to savolitinib plus osimertinib [126]. The response rate in patients progressing on osimertinib was 30%. On the basis of the findings, two phase II trials are ongoing with savolitinib in patients with EGFR mutant-NSCLC with *MET* amplification and progression on previous osimertinib (SAVANNAH (NCT03778229) and ORCHARD (NCT03944772) trials). The phase II INSIGHT 2 study (NCT03940703) is currently investigating tepotinib plus osimertinib in patients with EGFR-mutant NSCLC with acquired resistance to prior EGFR TKIs due to *MET* amplification.

Recent data demonstrate promising activity of the combination of amivantamab, an EGFR-*MET* bispecific antibody, and lazertinib, a third-generation TKI, in both treatment-naïve and osimertinib-relapsed patients with EGFR mutated NSCLC [127]. Osimertinib-resistance mutations or amplifications in EGFR/*MET* were identified by NGS in both liquid and tumour biopsies. Of seventeen patients with EGFR and/or *MET*-based resistance, the ORR was 47%, median DOR was 10.4 months, clinical benefit response rate was 82%, and median PFS was 6.7 months. IHC staining for EGFR and *MET* expression was explored as a potential biomarker for response. Among ten patients whose tumours stained high for EGFR and *MET* expression, 90% had a tumour response. Additional validation analyses with both NGS and IHC are needed to confirm these promising preliminary data and to identify patients most likely to benefit from the amivantamab and lazertinib combination.

### Recommendations

29. Patients with advanced EGFR-mutated NSCLC progressing on first and second generation EGFR TKIs without a T790M resistance mutation or patients progressing on osimertinib regardless of line of therapy and who have a *MET* amplification should be considered for clinical trials evaluating *MET* inhibitors.

## 9. What Are Potential Strategies to Overcome Resistance to *MET* Inhibitors?

Resistance mechanisms to *MET* TKIs are not well characterized. Acquired *MET* kinase domain mutations in residues D1228 and Y1230 confer resistance to type I *MET* TKIs in vitro by weakening chemical bonds between the drug and the *MET* kinase domain [128,129]. In addition, the solvent front G1163R mutation confers in vitro resistance to crizotinib but not to type Ib *MET* inhibitors like tepotinib, capmatinib, and savolitinib [130].

Analysis of twenty samples from patients with *MET*ex14 NSCLC with acquired resistance to a *MET* TKI revealed on-target resistance mediated by secondary *MET* kinase domain mutations and/or amplification in seven patients [131]. Off-target resistance, resulting from the activation of bypass signalling due to amplification of the ERBB family of receptor tyrosine kinase genes (BRAF amplification, KRAS amplification, and KRAS mutations), was detected in nine patients. One case displayed both on- and off-target mechanisms of resistance. In two patients with on-target resistant mutations, switching between type I and type II *MET* TKIs resulted in partial responses.

Data indicate that capmatinib has modest activity in crizotinib-pretreated *MET*-altered NSCLC [132]. Two out of twenty patients (fifteen patients with *MET*ex14 skipping mutation and five with *MET* amplification) achieved a partial response to capmatinib, and fourteen had SD, yielding a DCR of 80%. Both patients with a partial response had received chemotherapy with pembrolizumab as intervening therapy between crizotinib and capmatinib. Among five patients who discontinued crizotinib for intolerance, DCR was 83%, including two patients with best tumour shrinkage of −25% and −28%. Intracranial DCR among four patients with measurable brain metastases was 100%, with no observed intracranial objective responses. Overall, median PFS and OS were 5.5 months and 11.3 months, respectively. *MET* D1228 and Y1230 mutations and MAPK alterations were recurrently detected in post-crizotinib, pre-capmatinib plasma. The mutations persisted in plasma during treatment with capmatinib. New and persistent *MET* mutations and MAPK pathway alterations were detected in plasma at progression while on capmatinib, suggesting that capmatinib cannot easily overcome the secondary mutations.

In preclinical studies, class II *MET* TKIs (e.g., cabozantinib, merestinib, glesatinib) that bind to *MET* in a configuration that does not rely on interactions with the activation loop retained activity against *MET* D1228 and Y1230 mutations [58]. These observations have been confirmed by clinical case reports [133]. Ongoing studies with merestinib (NCT02920996) and cabozantinib (NCT03911193) in *MET* TKI-pretreated patients may help answer whether preclinical sensitivity consistently translates into clinical response.

Based on current evidence, in the context of on-target resistance, additional *MET*-targeted strategies employing *MET* antibodies or *MET* antibody–drug conjugates should be explored prospectively through clinical studies [134,135,136]. Initial data from the phase I CHRYSALIS study evaluating amivantamab in patients with *MET*ex14 skipping mutations showed anti-tumour activity in treatment-naïve and previously-treated patients, including patients previously treated with *MET* inhibitors [137]. For off-target mutations, combining *MET* inhibitors with another targeted therapy seems to be an appropriate approach that requires further study.

### Recommendations

30. Patients with advanced NSCLC *MET* alterations (either *MET*ex14 skipping and amplifications) resistant to *MET* inhibitors should be encouraged to enroll in clinical trials whenever possible. Patients progressing on type I *MET* inhibitors may be candidates for clinical trials with type II *MET* inhibitors or combination therapies.
31. There is currently no evidence that resistance profiling of the tumour post-*MET-*targeted therapy impacts patient outcomes; therefore, it is not recommended outside of clinical trials.

## 10. Conclusions

Lung cancer patients presenting with *MET* alterations represent a diverse population regarding their clinical presentation, underlying histology, and genomic instability. As with other driver mutations, timely detection and initiation of appropriate treatment are key to optimizing outcomes. *MET* inhibitors are emerging as effective and safe options for patients with *MET*ex14 skipping mutations; however, their sequencing, among other available options, requires further study. Participation in clinical trials is recommended for patients with *MET* amplification because of unanswered questions including optimal assays and cut-offs, as well as other clinicopathological characteristics of these patients that might be implicated in therapeutic decision making. Upcoming results from ongoing clinical trials and the emergence of novel agents and combinations will further streamline the management of patients with *MET*-altered NSCLC.

## Figures and Tables

**Table 1:** TKIs Targeting *MET*ex14 skipping mutations.

Compound	Targets	Type of Inhibitor	Enzyme IC^50^, nM	Cellular IC^50^ (Cell Line), nM	Clinicaltrials.gov NCT Number
Crizotinib	*MET, ALK, ROS1*	Type Ia	<1.0	8 (A549)	NCT00585195 (PROFILE-1001)
					NCT02465060 (NCI-MATCH)
					NCT02499614 (METROS)
					NCT02664935 (Matrix)
Capmatinib	*MET*	Type Ib	0.13	0.4 (H596)	NCT02750215
				0.7 (A549)	NCT01324479
Tepotinib	*MET*	Type Ib	3	9 (EBC-1)	NCT02864992 (VISION)
Savolitinib	*MET*	Type Ib	5	4 (H1993)	NCT02897479
Bozitinib	*MET*	Type I	8	5.8 (LU1901)	NCT03175224
				17 (LI0612)	NCT01639508
Cabozantinib	*MET, VEGFR2, RET, KIT, TIE-2, AXL*	Type II	1.3	7.8 (PC3)	NCT02544633
Glesatinib	*MET, VEGFR, RON, TIE-2*	Type II	1	20 (MKN45)	NCT02920996
Merestinib	*MET, TIE-1, AXL, ROS1, DDR1/2, FLT3, MERTK, RON, MKNK1/2*	Type II	4.7	35 (H460) 52 (S114)	NCT02897479

*ALK*, anaplastic lymphoma kinase; *DDR1/2*, discoidin domain receptor tyrosine kinase 1/2; *FLT3*, FMS-like tyrosine kinase 3; IC^50^, half inhibitory concentration; *MERTK*, *MER* receptor tyrosine kinase; *MET*, mesenchymal–epithelial transition; *MKNK1/2*, mitogen-activated protein (MAP) kinase-interacting serine/threonine-protein kinase 1/2; NCT, national clinical trial; TKIs, tyrosine kinase inhibitors.

**Table 2:** Efficacy of Type Ib *MET* inhibitors in metastatic NSCLC with *MET*ex14 skipping mutations.

Drug	Cohort	ORR, % (95% CI)	mDOR, Months (95% CI)	mPFS, Months (95% CI)	mOS, Months (95% CI)
Tepotinib	Overall (*n* = 146)	45.2 (37.0, 53.6)	11.1 (8.4, 18.5)	8.9 (8.2, 11.0)	17.6 (15.0, 21.0)
	1st line (*n* = 65)	44.6 (32.3, 57.5)	10.8 (6.9, NE)	8.5 (5.5, 11.3)	16.3 (9.7, 29.7)
	2nd line (*n* = 47)	46.8 (32.1, 61.9)	12.4 (9.5, NE)	9.5 (6.9, 13.7)	19.9 (15.0, 25.8)
	≥2nd line (*n* = 81) ^b^	45.7 (34.6, 57.1)	11.1 (0.5, 18.5)	10.9 (8.2, 12.7)	19.7 (15.0, 21.0)
Capmatinib	Overall (cohorts 4, 5b, 6, 7; *n* = 160)	52.5 ^a^	NR	NR	NR
	1st line (cohort 5b; *n* = 28)	67.9 (47.6, 84.1)	12.6 (5.6, NE)	12.4 (8.2, 23.4)	20.8 (12.4, NE)
	1st line (cohort 7; *n* = 32)	65.6 (46.8–81.4)	NE ^d^ (5.5–NE)	10.8 ^d^ (6.9–NE)	NE ^‡^ (10.6–NE)
	2nd line (cohort 6; *n* = 31)	51.6 (33.1, 69.8)	8.4 (4.2, NE)	6.9 (4.2, 13.3)	NR
	≥2nd line (cohort 4; *n* = 69 ^c^)	40.6 (28.9, 53.1)	9.7 (5.6, 13.0)	5.4 (4.2, 7.0)	13.6 (8.6, 22.2)
Savolitinib	Overall (*n* = 70)	42.9 (31.1–55.3)	8.3 (5.3–16.6)	6.8 (4.2–9.6)	NR
	PSC (*n* = 25)	40.0 (21.1–61.3)	17.9 (4.1–NE)	5.5 (2.8–6.9)	NR
	Other NSCLC (mainly adenocarcinoma; *n* = 45)	44.4 (29.6–60.0)	8.3 (4.2–9.7)	6.9 (4.2–13.8)	NR
	1st line (*n* = 28)	46.4 (27.5–66.1)	5.6 (4.2–16.6)	5.6 (4.1–9.6)	NR
	Previously treated (*n* = 42)	40.5 (25.6–56.7)	9.7 (4.9–NE)	6.9 (4.1–19.3)	NR

CI, confidence interval; mDOR, median duration of response; *MET*, mesenchymal–epithelial transition; mOS, median overall survival; mPFS, median progression-free survival; NE, not estimable; NR, not reported; NSCLC, non-small-cell lung cancer; ORR, overall response rate; PSC, pulmonary sarcomatoid carcinoma; ^‡^, Not yet mature; ^a^ Data not reported. Manually calculated from 1 CR, 18 PRs in Cohort 5b (1st line), 21 PR in Cohort 7 (1st line), 28 PRs in Cohort 4 (≥2nd line) and 16 PRs in Cohort 6 (2nd line); ^b^ 47 patients received tepotinib as second-line (58%); ^c^ 51 patients received capmatinib as second-line (73.9%); ^d^ Data not mature at the data cutoff date.

**Table 3:** Trials with *MET* inhibitors in NSCLC with *MET* amplification.

Drug	Trial	*MET* Amplification Cut Offs	*n*	Type of Biopsy	ORR, % (95% CI)	Median DOR Months (95% CI)	Median PFS Months (95% CI)
Crizotinib	PROFILE 1001	*MET/CEP7* ratio: ≥ 4—High	21	Tumour tissue	38 (18.1–61.6)	5.2 (3.3–25.8)	6.7 (3.4–9.2)
		*MET/CEP7* ratio: >2.2 to <4—Medium	14		14.3 (1.8–42.8)	3.8 (3.8–3.8)	1.9 (1.3–5.6)
		*MET/CEP7* ratio: ≥1.8 to ≤2.2—Low	3		33 (0.8–90.6)	12.2 (12.2–12.2)	1.8 (0.8–14.0)
		GCN ≥ 6	15		40% ^a^	4.86–12.02 ^b^	0.85–14.9 ^b^
Capmatinib	GEOMETRY-mono-1	Cohort 1a: GCN ≥ 10	69	Tumour tissue	29 (19–41)	8.3 (4.2–15.4)	4.1 (2.9–4.8)
		Cohort 1b: GCN 6 to 9 ^c^	42		12 (4–26)	24.9 (2.7–24.9)	2.7 (1.4–3.1)
		Cohort 2: GCN 4 or 5 ^c^	54		9 (3–20)	9.7 (4.2–NE)	2.7 (1.4–4.1)
		Cohort 3: GCN < 4 ^c^	30		7 (1–22)	4.2 (4.2–4.2)	3.6 (2.2–4.2)
Tepotinib	VISION	Cohort B: *MET* GCN >2.5	24	Liquid biopsy	41.7 (22.1, 63.4)	NE (2.8, NE)	4.2 (1.4, NE)

NE, not estimable; ^a^ 95% CI not reported; ^b^ Median not reported; ^c^ Closed for futility.

## Data Availability

The data discussed herein are available within the article and its references.
